# Laparoscopic Treatment of a Spontaneously Ruptured Kidney (Wunderlich Syndrome)

**DOI:** 10.1155/2015/701046

**Published:** 2015-02-26

**Authors:** Katharina Maria Bretterbauer, Dean Markić, Daniela Colleselli, Stephan Hruby, Ahmed Magdy, Günter Janetschek, Michael Josef Mitterberger

**Affiliations:** ^1^Department of Urology, University Clinics Salzburg, 5020 Salzburg, Austria; ^2^Department of Urology, University Hospital Rijeka, 51000 Rijeka, Croatia

## Abstract

Spontaneous, nontraumatic retroperitoneal hemorrhage or Wunderlich syndrome (WS) is a rare but potential life-threatening condition. In most patients a bleeding renal neoplasm is the cause of the retroperitoneal hematoma. The management of this condition includes a conservative approach in the hemodynamically stable patients and active treatment in the unstable patients. Active treatment includes angioembolization or surgery. If angioembolization is not available open surgery is in most cases the preferred approach. We present a patient with a spontaneously ruptured kidney due to a central renal angiomyolipoma, which was treated by laparoscopic nephrectomy.

## 1. Introduction

Spontaneous retroperitoneal hemorrhage or Wunderlich syndrome (WS) is a relatively uncommon cause of acute abdominal pain. It can occur for a number of reasons, the most common from bleeding renal neoplasms, especially renal angiomyolipoma (AML) [1]. Symptoms of WS can range from mild to life-threatening. Although laparoscopic nephrectomy has been implemented as routine surgical approach for benign and malignant renal tumours as elective procedure, it is normally not used in emergency situations as primary treatment approach. We present a case of a ruptured kidney due to a central renal AML in a female patient treated effectively by laparoscopy.

## 2. Case Report

**Figure 1:**
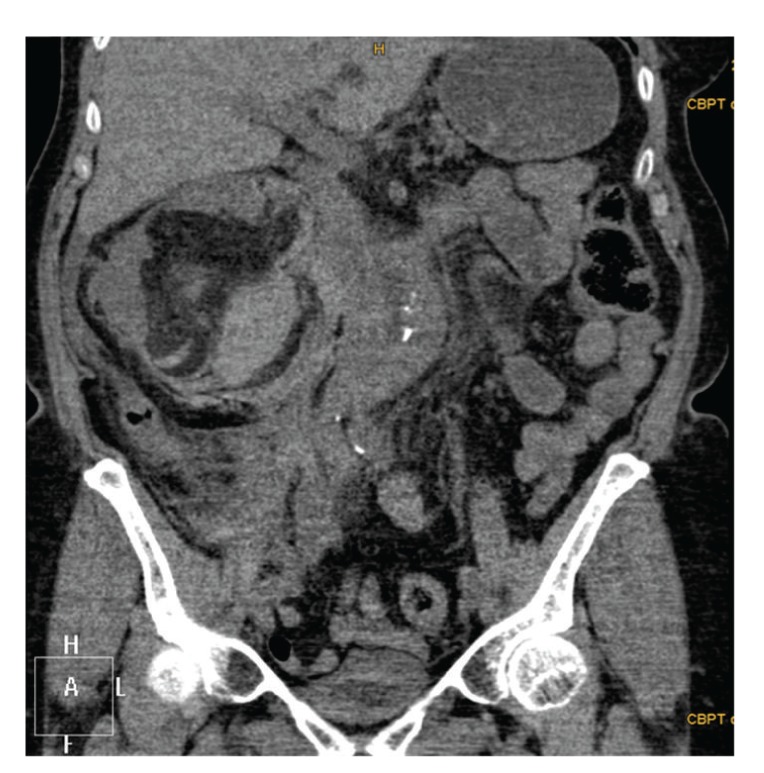
Computerized tomography of the abdomen demonstrating spontaneous retroperitoneal hemorrhage and central renal angiomyolipoma of the right ruptured kidney. 110 × 111 mm (150 × 150 DPI).

**Figure 2:**
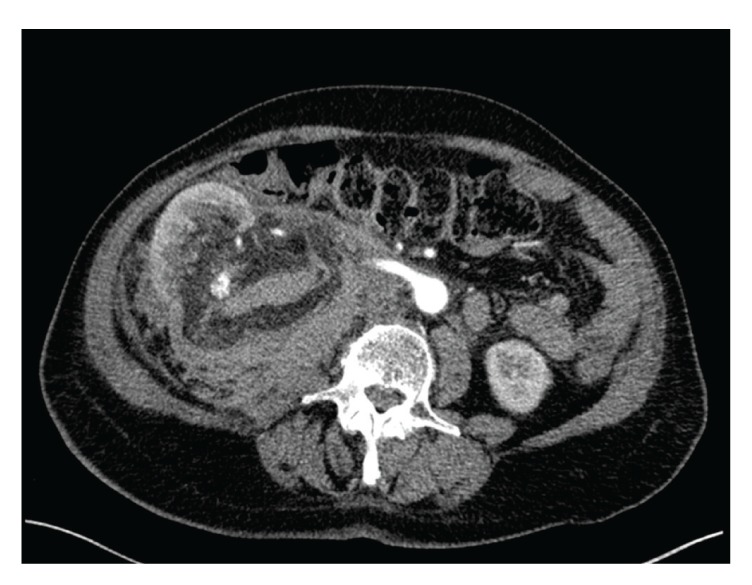
Arterial phase of the computerized tomography showing the ruptured kidney with the central renal angiomyolipoma with retroperitoneal hematoma and precaval right renal artery. 116 × 87 mm (150 × 150 DPI).

A 74-year-old female patient presented herself at the emergency room because of sudden and now constant pain in the right lumbar region. Physical examination was normal except mild tenderness in the right lumbar region. The patient denied any trauma and had no premedication. Initial hemoglobin level was 10.2 g/dL, hematocrit 29%, creatinine value 0.7 mg/dL, and eGFR >70 mL/min. The urine showed no macroscopic hematuria. The ultrasound (US) examination showed a hypoechogenic mass around the right kidney and a 10 cm central renal lesion. The consecutive computerized tomography (CT) revealed ruptured kidney due to a 10 cm central renal AML with bleeding in the tumour and a large perinephric hematoma around the right kidney (Figure 1). The hematoma was confined by Gerota's fascia and also a single precaval right renal artery was noticed (Figure 2).

The findings of US and CT established the diagnosis of a spontaneous ruptured central renal AML with significant hematoma and rupture of the right kidney. Following blood tests showed a significant drop in the hemoglobin level to 7.9 g/dL; the patient got hemodynamically unstable and therefore a surgical therapy became necessary.

**Figure 3:**
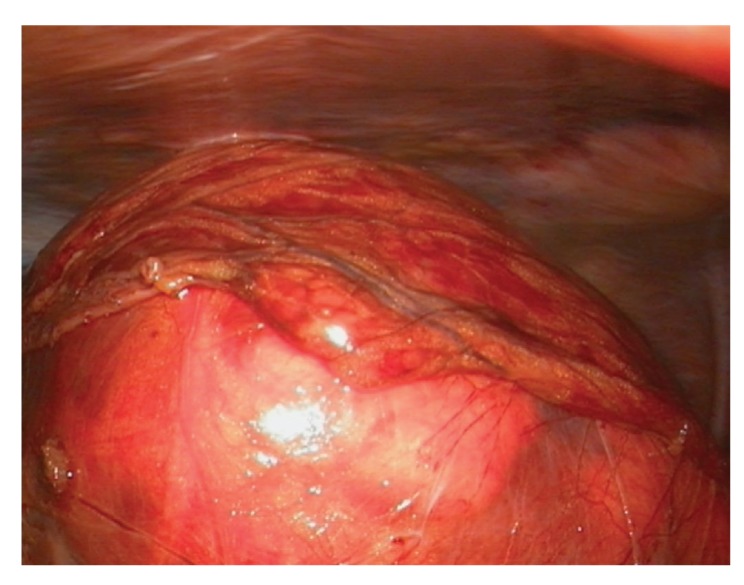
Intraoperative view of large retroperitoneal hematoma and initial incision of parietal peritoneum above the hematoma. 160 × 122 mm (116 × 117 DPI).

We choose the transperitoneal laparoscopic approach. The following briefly describes the operation. Under prophylactic antibiotic therapy and after induction of general anesthesia the patient was placed in the lateral position. Surgery was done transperitoneally in a standard technique for nephrectomy using four ports (three 11 mm and one 5 mm) and a camera with a 30-degree lens. Immediately after entrance in the peritoneum a large retroperitoneal hematoma was seen (Figure 3). The colon was displaced by the hematoma. The operation was similar to standard nephrectomy. Briefly, the right colon was mobilized medially. The second portion of the duodenum was dissected and the anterior surface of the vena cava was exposed. The renal vessels were isolated and clipped with Hem-o-lok (Teleflex Medical, USA) and an adequate hemostasis was achieved. During the entire procedure there was persistent bleeding from the hematoma's surface. The specimen was placed in a laparoscopic entrapment bag and removed. Finally, the drain was placed and the port sites were closed. The blood loss during operation was 750 mL (including the hematoma blood content). The duration of operation was 120 minutes. No intraoperative complications were encountered. The specimen was sent to the uropathologist. Final histopathology showed a central renal AML without malignancy and with highest diameter of 10 cm.

The postoperative course was uneventful. The drain was removed three days after operation. During the stay in the hospital the patient received prophylactic antibiotic (1 gram of amoxicillin/clavulanic acid twice a day) and low-molecular weight heparin therapy. Postoperative hemoglobin after two doses of blood was 10.2 g/dL, hematocrit 29%, creatinine 1.10 mg/dL, and eGFR 51 mL/min. The patient was discharged five days after operation.

## 3. Discussion

Renal AMLs are infrequent, benign lesions consisting of three different tissue lines: smooth muscle cells, blood vessels, and adipose tissue. Usually AML is an incidental finding discovered during routine radiological studies. AML is in most cases sporadic and typically is seen in the middle-aged patients. It is often solitary and more common in the women and on the right kidney. Approximately 20% of AMLs occur in a hereditary form in patients with tuberous sclerosis syndrome. Zhang et al. analyzed 165 patients with WS and revealed that renal neoplasms were the most common cause of WS (61.5%), compared to vascular diseases (17%), infection (2.4%), miscellaneous (12.7%), and idiopathic (6.7%) [1]. The most common cause was AML (29.1%), renal cell cancer (26.1%), and polyarteritis nodosa (12.1%).

Symptomatic patients may present with Lenk's triad: flank pain, palpable tender mass, and macrohematuria [2]. Massive retroperitoneal hemorrhage from AMLs can be found in up to 10% of patients and represent the most significant and feared complications [3]. Larger lesions (>4 cm) have a higher incidence of spontaneous bleeding [4]. The diagnosis is mainly based on imaging techniques.

Management is dictated by the clinical condition of the patient and by the underlying aetiology. Some authors argue that WS can be managed conservatively if the hemorrhage is self-limiting and the patient is responsive to fluid resuscitation. Others argue that selective arterial embolization is the preferred treatment modality because it is a nephron sparing procedure.

Active surveillance for renal AML is a valuable option in some patients. In the patients under active surveillance the risk factor for delayed intervention included tumor size >4 cm and symptoms at diagnosis. Selective angioembolization was the first-line option used for active treatment after active surveillance was discontinued [5].

For patients who are clinically unstable, surgery is a mandatory option [6]. In patients with ruptured AML the preferred treatment was nephrectomy (68.4% patients). Evacuation of hematoma was done in 10.3% of patients, partial nephrectomy in 4.8% of patients, embolization in 4.2% of patients, and conservative treatment in 9.7% patients [1].

In most of the cases of ruptured AML nephrectomy was done by open surgery [1]. The development of minimal invasive procedures in the last two decades established laparoscopic and robot-assisted nephrectomy as standard procedures for the elective nephrectomy. Possible use of these techniques in the emergency settings is still under consideration. In the literature we found few descriptions of using minimal invasive procedures for treatment of a ruptured kidney due to an AML.

The first reported case about laparoscopic management of WS was published in 2011 [7]. In patient with ruptured AML laparoscopic nephrectomy was done in the acute phase. Operation was performed transperitoneally with standard positions of trocars. Surgical time was 250 minutes and blood loss 2000 mL (including hematoma). Peña et al. presented four patients with WS treated by laparoscopy [7]. The cause of the bleeding was AML in two patients and papillary renal cell cancer in two patients. All patients were first treated conservatively and laparoscopic nephrectomy was performed 3–6 months after haemorrhage. In one patient with AML partial laparoscopic nephrectomy was made. Intraoperatively authors found significant fibrosis involving ipsilateral kidney and surrounded tissue and concluded that a delayed procedure, due to fibrosis and associated tissue plane loss, is a technically challenging operation. Recently Ploumidis et al. published their experience with robotic-assisted laparoscopic partial nephrectomy for ruptured AML [8]. The AML was 7.6 cm in greatest diameter and partial nephrectomy was successfully made with warm ischemia time of 20 minutes.

In the present case report the laparoscopic procedure was performed in the acute phase of WS. The patient was hemodynamically unstable and the ruptured kidney due to the large central AML was the reason for the surgery and for the total nephrectomy. If the AML would have been smaller and exophytically located, partial nephrectomy would have been considered as in the elective cases. Laparoscopy provides all known benefits of minimally invasive procedures. For instance, the pressure induced by the pneumoperitoneum may help in diminishing bleeding from the hematoma during dissection [7]. The operation in WS is an imitation of the standard laparoscopic nephrectomy, but the massive retroperitoneal hematoma and bleeding from the hematoma during the dissection makes the whole operation more challenging. Therefore it is essential that the operation is performed by an experienced surgeon. In the case of difficulties during the laparoscopic procedure the operation should be converted to open surgery.

## 4. Conclusion

Laparoscopic nephrectomy in a spontaneous retroperitoneal hemorrhage (Wunderlich Syndrome) due to a large central renal AML is feasible and safe. The operation should be performed by a skilled surgeon and in selected patients only.

## References

[1] Zhang J. Q., Fielding J. R., Zou K. H. (2002). Etiology of spontaneous perirenal hemorrhage: a meta-analysis. *Journal of Urology*.

[2] Simmons J. L., Hussain S. A., Riley P., Wallace D. M. A. (2003). Management of renal angiomyolipoma in patients with tuberous sclerosis complex. *Oncology Reports*.

[3] Moratalla M. B. (2009). Wunderlich's syndrome due to spontaneous rupture of large bilateral angiomyolipomas. *Emergency Medicine Journal*.

[4] Maclean D. F. W., Sultana R., Radwan R., McKnight L., Khastgir J. (2014). Is the follow-up of small renal angiomyolipomas a necessary precaution?. *Clinical Radiology*.

[5] Ouzaid I., Autorino R., Fatica R. (2014). Active surveillance for renal angiomyolipoma: outcomes and factors predictive of delayed intervention. *BJU International*.

[6] Sparks D., Chase D., Thomas D., Arnott J. (2011). The Wunderlich's syndrome secondary to massive bilateral angiomyolipomas associated with advanced tuberous sclerosis. *Saudi Journal of Kidney Diseases and Transplantation*.

[7] Peña J. A., Serrano M., Cosentino M. (2011). Laparoscopic management of spontaneous retroperitoneal hemorrhage. *Urologia Internationalis*.

[8] Ploumidis A., Katafigiotis I., Thanou M., Bodozoglou N., Athanasiou L., Ploumidis A. (2013). Spontaneous retroperitoneal hemorrhage (Wunderlich syndrome) due to large upper pole renal angiomyolipoma: does robotic-assisted laparoscopic partial nephrectomy have a role in primary treatment. *Case Reports in Urology*.

